# Stem cells for treatment of liver fibrosis/cirrhosis: clinical progress and therapeutic potential

**DOI:** 10.1186/s13287-022-03041-5

**Published:** 2022-07-26

**Authors:** Pinyan Liu, Yongcui Mao, Ye Xie, Jiayun Wei, Jia Yao

**Affiliations:** 1 The First Clinical Medical College of Lanzhou University, Lanzhou, China; 2 Key Laboratory of Biotherapy and Regenerative Medicine of Gansu Province, Lanzhou, China

**Keywords:** Stem cells, Liver fibrosis/cirrhosis, Clinical trials, Cell therapy, Mechanism

## Abstract

Cost-effective treatment strategies for liver fibrosis or cirrhosis are limited. Many clinical trials of stem cells for liver disease shown that stem cells might be a potential therapeutic approach. This review will summarize the published clinical trials of stem cells for the treatment of liver fibrosis/cirrhosis and provide the latest overview of various cell sources, cell doses, and delivery methods. We also describe the limitations and strengths of various stem cells in clinical applications. Furthermore, to clarify how stem cells play a therapeutic role in liver fibrosis, we discuss the molecular mechanisms of stem cells for treatment of liver fibrosis, including liver regeneration, immunoregulation, resistance to injury, myofibroblast repression, and extracellular matrix degradation. We provide a perspective for the prospects of future clinical implementation of stem cells.

## Introduction

Liver diseases cause about two million deaths worldwide each year, accounting for 3.5% of all annual deaths, of which one million die from complications of liver cirrhosis [1]. Liver cirrhosis is the 11th most common cause of death worldwide, and the number and proportion of global deaths with this cause will continue to increase in the future (more than 1.32 million died of cirrhosis in 2017 but less than 899,000 deaths in 1990 [2]). Chronic liver diseases are generally divided into four stages according to severity: (1) inflammation; (2) liver fibrosis; (3) cirrhosis; and (4) end-stage liver disease (ESLD) or liver cancer [3]. This is often a consecutive process in which liver function of advanced cirrhosis or ESLD is irreversibly impaired; the only curative treatment is orthotropic liver transplantation (OLT). It is high on the agenda that we should explore new therapeutic strategies because of the major limitations of OLT, such as immune rejection, adverse post-operative complications, organ shortages, etc. In 1976, the first preclinical primary human hepatocytes (PHHs) transplantation shed light on the prospect of cell therapy [4]. Since then, cell therapy for chronic liver diseases has developed rapidly. PHHs are regarded as an ideal source to treat metabolic liver disease and have been set into clinical use [5]. However, PHHs are so hard to expand in vitro and their sources are so confined, that people are forced to turn to additional cell sources. Currently, evidence that stem cells are effective for the treatment of liver fibrosis and cirrhosis in phase I and II clinical trials has intensified attention, in hopes that stem cell therapy can reverse liver fibrosis and cirrhosis. Due to the ethical issues of the extraction and application of human primary stem cells, the number of clinical trials using stem cells for the treatment of liver fibrosis and cirrhosis is limited. Over the past few decades, the effectiveness, feasibility and safety of stem cell therapies in animal models have been extensively reported in the literature. Animal models bridge the translation from basic research to clinic application. Inevitably, rodent models are inconsistent with human diseases, and it is necessary to test safety and efficacy in animal models closer to humans before clinical trials [6, 7]. More importantly, due to the difficulty in obtaining clinical specimens, the current stem cell therapy for human liver diseases can only obtain the outcome of the patient. The mechanism of stem cell therapy is based on animal models. The mechanism of action for donor stem/progenitor cells has been previously summarized in two major pathways: (1) differentiation into functional cells to replace damaged cells; (2) producing bioactive factors that enhance patient’s own tissue-resident progenitor cells proliferation or maturation and immunoregulatory factors that modulate the progression of inflammation. While the first one would realistically require several weeks to be completed and insufficient to support liver function or restore the organ, the second one has been largely recognized as prominent and efficient. Moreover, in fibrotic diseases, the extracellular matrix (ECM) remodeling mediators secreted by stem cells such as matrix metalloproteinase (MMP), and tissue inhibitor of metalloproteinase (TIMP) have been reported instrumental to fibrosis reversal. Compared with conventional treatment options, stem cell-based therapies are less invasive in patients than surgical methods and carry a low risk of immune rejection. In this review, we summarize the latest clinical developments in the field of using stem cells for the management of liver fibrosis and cirrhosis in the hope that they will provide insight into the subsequent clinical translation of stem cells.

## Clinical progress using stem cells for liver cirrhosis/fibrosis therapy

**Fig. 1:**
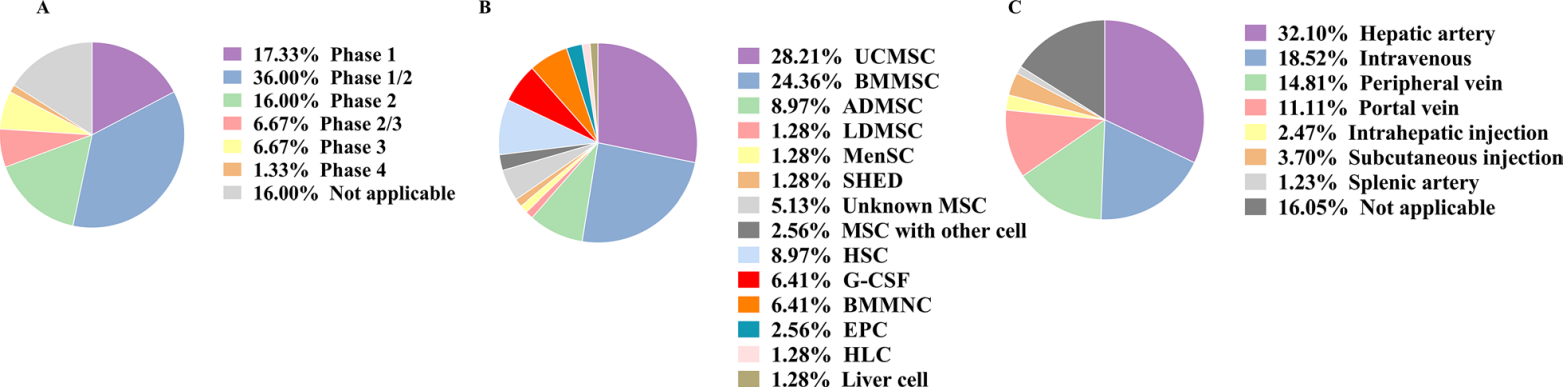
Classification of registered stem cell-related clinical trials in liver fibrosis or cirrhosis. **A** Clinical trials classified by clinical phase; **B** different stem cell applied in current clinical trials; UCMSC, umbilical cord mesenchymal stem cell; BMMSC, bone marrow mesenchymal stem cell; ADMSC, adipose-derived mesenchymal stem cell; LDMSC, liver-derived mesenchymal stem cell; MenSC, menstrual blood-derived mesenchymal stem cell; SHED, stem cells from human exfoliated deciduous teeth; HSC, hematopoietic stem cell; G-CSF, granulocyte-colony stimulating factor; BMMNC, bone marrow mononuclear cell; EPC, endothelial progenitor cell; HLC, hepatocyte-like cell; **C** clinical trials classified by delivery route. All of data collected from www.clinicaltrials.gov

When clinicaltrials.gov was searched for the condition “liver diseases” and other terms such as “stem cells”, a total of 160 clinical trials using stem cells for the treatment of liver diseases, including end-stage liver disease, liver cancer, liver fibrosis, and cirrhosis, were found registered, of which 75 trials (47%) focus on liver cirrhosis or fibrosis (excluding terminated and withdrawn trials). In these 75 cases, most trials concentrated on Phase I/II (36%) (Fig. 1A).

For cell therapy, the following are of particular concern: cell type, cell number, and route of administration.

The cell sources used to treat liver fibrosis or cirrhosis are diverse, with the most widely used being mesenchymal stem cells (MSCs) (73%) (Fig. 1B). The MSCs currently being applied in clinical trials are obtained from different tissues such as bone marrow, umbilical cord, adipose, menstrual blood, dental pulp, and liver. Moreover, meta-analyses show that stem cells derived from bone marrow are more effective than those derived from the umbilical cord, and superior improvement in model for end-stage liver disease (MELD) score, albumin (ALB) levels, alanine aminotransferase (ALT) levels, and total bilirubin (TBil) occurred in the bone marrow mesenchymal stem cell (BMMSC) group than umbilical cord mesenchymal stem cell (UCMSC) group [8]. This may be due to differences in the adhesive and migratory or homing properties of MSCs isolated from different tissue. The fate of cells injected into the circulation and the capacity of the cells to promote repair and immune regulation at the site of damage are affected by both properties. USMSC appears to adhere and migrate more rapidly from flow than BMMSC, but BMMSC surpasses USMSC in terms of spreading kinetics [9]. More vitally, we have no way of telling who homed better to the liver, UCMSC or BMMSC? Other possible explanations included BMMSC cleared bacteria and enhanced cell survival, and the superior regulation of the pro/anti-inflammatory response compared to UCMSC [10]. In a previous study, transplantation of MSC enhanced engraftment of hematopoietic stem cell (HSC) [11]. Ghavamzadeh et al. [12] demonstrated that co-transplantation of HSC with MSC increased the rate of replacement of recipient hepatocytes by donor-derived cells and alleviated liver fibrosis. But the most recent research showed that MSC co-transplantation with HSC or Treg is feasible, but this approach couldn’t significantly improve liver fibrosis than HSC infusion only. Robust evidence that the MSCs used to support the donor's HSCs is of recipient origin [13]. In addition, intravenous MSCs have a short lifespan and cannot migrate out of the lungs [14]. Given this, MSC infusion after HSC transplantation may be more preferred than a single dose of MSC co-transplantation with HSC.

A cell count of 1 × 10^6^ or more is required for treatment. Cell dose depends on the patient weight, clinical situation, administration route, and cell type. In liver cirrhosis or failure, hepatocyte transplantation aims to provide functional parenchymal support to the damaged mass, bridging the recovery of the liver. Based on this concept, the cell count is roughly 10–15% of the theoretical liver mass, higher than liver metabolic disorders [15]. The maximum amount of cells injected is no more than 5% (approximately 1 × 10^11^ hepatocytes) of the liver parenchyma. The complications of cell infusion like portal hypertension and cell embolism limit the number of cells that can be infused [16]. Hepatocyte transplantation not only is inefficient but also needs to suppress the patient’s immune system. Local but not systemic administration of MSCs improved liver fibrosis after partial hepatectomy [17]. In the management of liver fibrosis, and cirrhosis, we found that the dose of MSCs was generally lower compared to that of hepatocytes, reflecting the fact that the dominant mechanism of action of MSC is not through replacement therapy, but rather a promotion of endogenous liver regeneration and immunomodulation mediated by secreted bioactive molecules. Within the range of safe and the lowest effective dosages for administration, there seems to be no difference in the outcome of the low, medium, and high dose groups [18]. Therapeutic effects of interval and repeated injections, such as twice per week, seem more stable and significant; however, a single and adequate injection is more effective [19].

Cell therapy routes are currently divided into two categories: infusion of suspension of stem cells or transplantation of the product of stem cells modified by bioengineering. Administration via the hepatic artery (32%) is the preferred choice for most clinical trials (Fig. 1C). This is probably because stem cells infused via the hepatic artery have a better ability to engraft than intravenous infusion [20]. Notably, of these clinical trials, only one used the bioengineered product that is an injectable collagen scaffold combined with hUCMSCs (NCT02786017).

Apart from the details in clinical trials, the safety and efficacy of cell therapy are most critical. Stem cells have made surprising progress in clinical trials for the treatment of cirrhosis or liver fibrosis. Common indicators available to evaluate the efficiency of stem cells are serum ALB level, serum bilirubin, Child–Pugh score, and MELD score. Safety is evaluated mainly in terms of postoperative adverse complications such as fever, anaphylaxis, cough, chest distress, and dyspnea, etc. Although these clinical trials seem safer, lager-scale studies are still in demand. Representative clinical trials using stem cells for the treatment of liver fibrosis or cirrhosis are shown in Table 1.

**Table 1:** Representative clinical trials using stem cells for the treatment of liver fibrosis or cirrhosis

Disease	No. of patients	Cell type	Dose	Route	Results	NCT number
Decompensated liver cirrhosis	219	Umbilical cord mesenchymal stem cells	0.5 × 10^6^ cells/kg	Infusion via intravenous (every 4 weeks, 3 cycles)	A significant decrease of ascites volume, increase of serum albumin level in patients and improvements in MELD score	NCT01220492
Alcoholic liver cirrhosis	12	Allogeneic bone marrow mesenchymal stem cells	5 × 10^7^ cells	Injection via hepatic artery at week 4, 8	Improvement in histology and Child–Pugh score, a significant decrease of levels of transforming growth factor-β1, type I collagen and α-smooth muscle actin	NCT01741090
Liver cirrhosis	6	Autologous adipose-derived mesenchymal stem cells	1 × 10^8^ cells/ml	Intrahepatic injection	Improvement in liver function, METAVIR score, Child–Pugh score, MELD score	NCT02297867
Liver fibrosis/Cirrhosis/Liver disease	50	Menstrual blood derived mesenchymal stem cells	1 × 10^6^ cells/kg	Infusion via intravenous (twice per week, for 2 weeks)	Not available in liver function improvement, ascites, Child–Pugh score, MELD score, SF36-quality of life, and complications	NCT01483248
Liver cirrhosis	9	Liver mesenchymal stem cells	-	-	Not available in blood parameters in tubing loop model and fibrin formation in thrombodynamics	NCT03632148
Decompensated liver cirrhosis	40	Stem cells from human exfoliated deciduous teeth	1 × 10^6^ cells/kg	Infusion via peripheral vein at week 0, 4, 8, 12	Not available in liver function, MELD score, Child–Pugh score, survival rate at half of one year, changes of life quality	NCT03957655
Liver cirrhosis/Liver failure	30	Hepatocyte-like stem cells	3–5 × 10^7^ cells	Injection via port vein	Decrease of volumes of ascites and prothrombin complex from international normalized ratio, increase of serum albumin, improvement in liver function, MELD score	NCT00420134
Liver cirrhosis	25	Fetal liver cells	5–10 × 10^8^ cells	Infusion via splenic artery	Improvements in follow-up Child–Pugh score and follow-up MELD score	NCT01013194
Liver cirrhosis	100	G-CSF	5 μg/kg	Injection via subcutaneously (every 12 h for 5 consecutive days, 4 cycles at 3 monthly intervals)	Increase in survival rate at 12 months, quality of life, HSC mobilization, improvement in MELD score, Child–Pugh score	NCT03415698
Decompensated fibrosis	27	Bone marrow-derived mononuclear cells and CD133^+^ cells	2–3 × 10^9^ mononuclear cells and 5–15 × 10^6^ CD133^+^ cells	Injection via port vein	A transient improvement in the MELD score was observed in CD133 + group and no difference in mononuclears group	NCT01120925
Alcoholic liver cirrhosis	5	Autologous CD34 + stem cells	1 × 10^6^–2 × 10^8^ cells	Injection via the portal vein or hepatic artery	3 of the 5 patients showed improvement in serum bilirubin and 4 of 5 in serum albumin	NCT00147043
Decompensated liver cirrhosis	14	Endothelial progenitor cells	1 × 10^8^ cells	Injection via hepatic artery	A significant improvement in MELD score and 5 of the 9 patients showed a decreased of hepatic venous pressure gradient	NCT01333228

## Stem cell types used for liver fibrosis/cirrhosis therapy

**Fig. 2:**
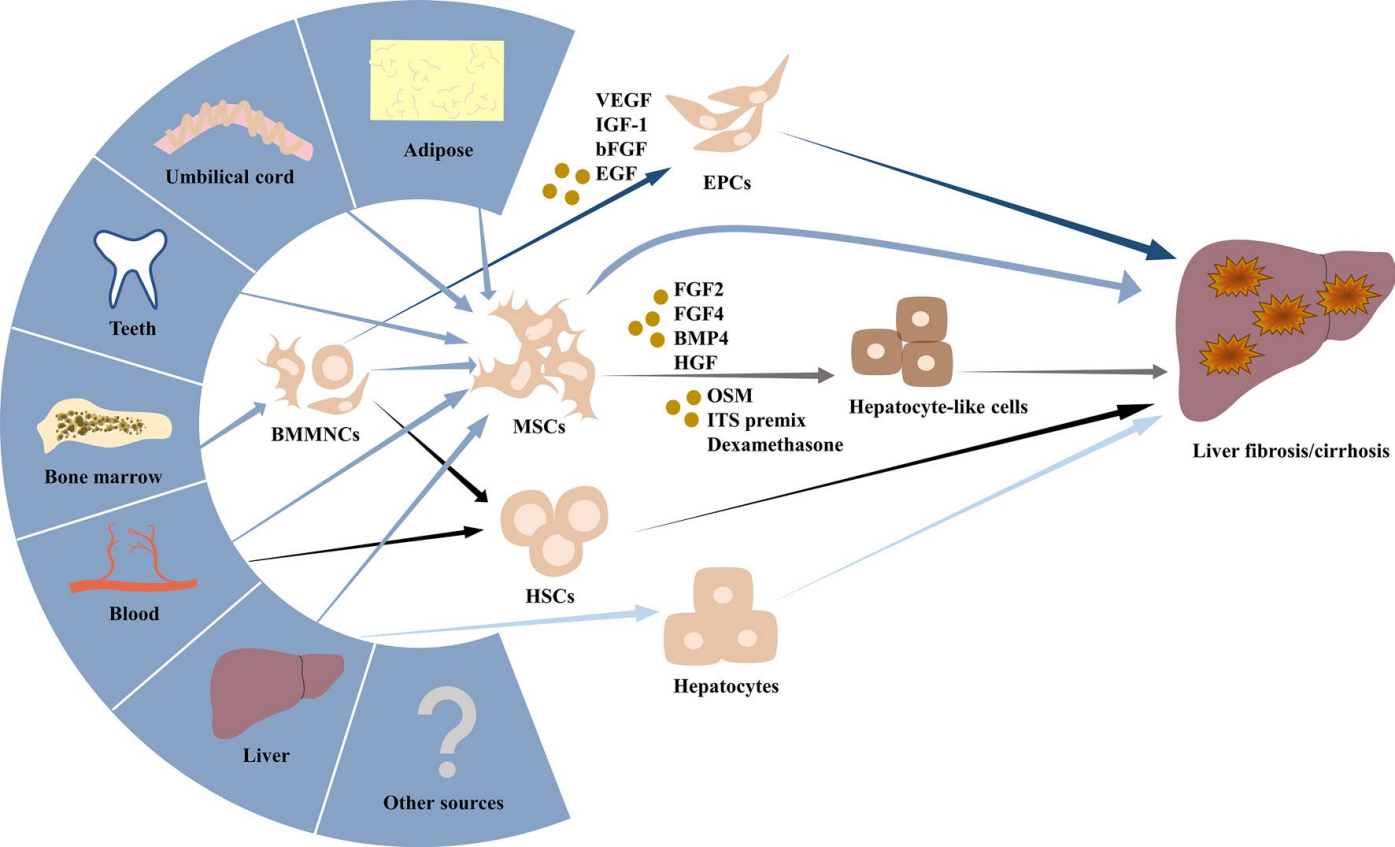
Candidate stem cell types for the treatment of liver fibrosis/cirrhosis. Different types of stem cells for the treatment of liver fibrosis/cirrhosis, especially MSCs, can originate from various tissues such as adipose, umbilical cord, teeth, bone marrow, blood, liver, etc. Differentiated hepatocyte-like cells and EPCs in vitro can also be used as alternative cell sources. MSC, mesenchymal stem cell; HSC, hematopoietic stem cell; BMMNC, bone marrow mononuclear cell; EPC, endothelial progenitor cell; FGF2, fibroblast growth factor 2; FGF4, fibroblast growth factor 4; BMP4, bone morphogenetic protein 4; HGF, hepatocyte growth factor; OSM, oncostatin M; ITS, Insulin-Transferrin-Selenium; VEGF, vascular endothelial growth factor; IGF-1, insulin-like factor-1; bFGF, basic fibroblast growth factor; EGF, epidermal growth factor

From the above clinical trials we learned that there is a wide selection of cell types that can be applied in the management of liver fibrosis and cirrhosis, and the following is a detailed description of the cell types mentioned (Fig. 2).

### Mesenchymal stem cells

MSCs are the most widely applied type of stem cells and can be obtained from every adult or perinatal tissue including common bone marrow, umbilical cord, adipose tissue, liver, teeth, and menstrual blood. In recent years MSCs were extracted from urine [21] and different parts of the placenta [22]. MSC has the same characteristics (positive for CD73, CD90, and CD105, and negative for CD34, CD14, or CD11b, CD79 or CD19, HLA-DR) [23].

#### Umbilical cord mesenchymal stem cells

UCMSCs can be obtained from Wharton’s jelly and cord blood. There are two general methods of obtaining harvesting cells: enzymatic digestion and the explants method. UCMSCs should be prepared in approved good manufacturing practice (GMP) [24].

hUCMSCs promoted liver repair and improved regenerative niche by secreting hepatocyte growth factor (HGF) and increasing the expression of proliferating cell nuclear antigen (PCNA) and Est-1 p42 protein in thioacetamide-injured mouse liver [25]. Moreover, hUCMSCs also demonstrated superior immunosuppressive capacity with high Treg promotion in a mouse model of liver disease [26]. Besides, hUCMSCs transplantation inhibits the activation of hepatic stellate cells by upregulating microRNA-455-3p to mediate p21-activated kinase-2 (PAK2) silence [27].

#### Bone marrow mesenchymal stem cells

BMMSCs are isolated from bone marrow mononuclear cells (BMMNCs). BMMNCs are seeded in a culture flask and then the non-adherent cells are removed, resulting in colonized BMMSCs [28].

Microencapsulated BMMSCs engrafted in damaged tissue have a protective effect since anti-apoptotic interleukin (IL)-6, insulin-like growth factor binding protein-2 (IGFBP-2), and anti-inflammatory IL-1 receptor antagonist (IL-1Ra) cytokines were released [29]. Besides, liver-specific ECM induced BMMSCs trans-differentiated to hepatic lineage and reversed liver fibrosis by replacing liver parenchymal cells to serve [30].

#### Adipose-derived mesenchymal stem cells

Adipose-derived mesenchymal stem cells (ADMSCs) can be obtained from liposuction aspirates or subcutaneous fat fragments and expanded in vitro. Adipose tissues were digested with collagenase, the resultant dispersed cells were collected and cultured in the flask [31].

The number of inflammation-infiltrated CD11b^+^ cells, Gr-1^+^ cells, and the ratio of CD8^+^/CD4^+^ decreased in steatohepatitis-induced cirrhosis with ADMSCs treatment [32]. Currently, ADMSCs and BMMSCs are considered to alleviate fibrosis through similar mechanisms [33].

#### Liver-derived mesenchymal stem cells

In short, mononuclear cells were isolated from liver fragments and cultured in vitro to obtain liver-derived mesenchymal stem cells (LDMSCs). Laboratories have successfully isolated human liver-derived mesenchymal-like stem cells, which highly express endodermal markers such as GATA4 and FOXA1. LDMSCs are positive for CD29, CD73, CD44, CD90, CD105, and CD166 together with, albumin, α-fetoprotein, cytokeratin 8, and cytokeratin 18 these hepatic markers [34]. LDMSCs both expressed some of the surface markers of MSC and possess some of the functions of hepatocytes, therefore indicating a partial commitment toward hepatic differentiation [35].

In thioacetamide-induced immunodeficient NRG mouse models with liver injury, hLDMSCs engrafted more rapidly in the damaged liver to differentiate into mature hepatocytes compared with hUCMSCs [36]. Furthermore, LDMSCs secrete higher levels of pro-angiogenic, anti-inflammatory, and anti-apoptotic cytokines compared to BMMSCs [34].

#### Menstrual blood-derived mesenchymal stem cells

Menstrual blood-derived mesenchymal stem cells (MenSCs) are isolated by density-gradient centrifugation from centrifuged human menstrual blood, they expressed MSC markers, but did not express HLA-DR, CD34, CD45, CD117. Interestingly, stage-specific embryonic antigen 4 (SSEA4) has not existed in MSCs from other sources, but SSEA4 expression in the MenSCs is controversial [37, 38].

MenSCs markedly decreased expression of alpha-smooth muscle actin (α-SMA) and transforming growth factor-β1 (TGF-β1) in liver tissue and suppressed activated hepatic stellate cells via paracrine mediators such as monocyte chemoattractant protein-1 (MCP-1), IL-6, HGF, etc, further degraded collagen content in carbon tetrachloride-induced liver fibrosis [39].

#### Stem cells from human exfoliated deciduous teeth

Stem cells from human exfoliated deciduous teeth (SHED) are isolated from remnant dental pulp tissues of healthy pediatric donors exfoliated deciduous teeth. There are differences in the properties of SHEDs obtained by different isolation methods [40]. They share MSC characteristics, including cell surface antigen expression and trilineage differentiation potential.

Transplanted SHED are capable of generating hepatocyte-like cells in vivo, under the stimulation of tumor necrosis factor-α (TNF-α), SHED-Heps showed cholangiogenic potential [41]. Moreover, SHED downregulated ECM resolution-associated mediators, such as MMP-2, MMP-9, TIMP-1, and TIMP-2 in the damaged liver [42]. In addition, SHED promoted recovery from hepatic fibrosis by preventing inflammatory infiltration [43].

### Hematopoietic stem cells

HSCs are primitive cells found in the bone marrow and blood. HSCs are purified from harvested peripheral blood mononuclear cells derived from patients who received subcutaneous injections of granulocyte-colony stimulating factor (G-CSF) or sorted from bone marrow through the flow sorting system. Besides, HSC can also be obtained from umbilical cord blood. Accordingly, HSCs are positive for CD34, CD45, and CD133 [44].

Two methods are available for the mobilization of stem cells from the bone marrow to the liver. One involves the isolation of stem cells from the marrow followed by their infusion into the body and the other is the administration of cytokines like G-CSF. Any drug with the capacity to mobilize bone marrow stem cells (BMSCs) can be used to treat liver fibrosis, but the degree of improvement may not be proportional to the efficiency of mobilization [45]. G-CSF mobilized BMSCs to reach the damaged liver for repair [46]. However, recent randomized clinical trials have shown that G-CSF cannot improve MELD scores in patients with compensated cirrhosis regardless of whether it is combined with HSCs, possibly due to adverse effects of G-CSF [44]. HSCs have negligible anti-inflammatory and antioxidant effects compared to BMMSC in liver injury [47]. HSCs could home to the damaged liver but barely converge toward the hepatic line. Their contribution to liver repair is mainly through the release of paracrine factors that stimulate endogenous hepatocyte regeneration and boost intrahepatic angiogenesis [48]. HSC transplantation or HSC co-transplantation with other stem cells has been shown to be feasible in refractory ascites with liver fibrosis [49] or patients with dedicator of cytokinesis 8 protein deficiency [50].

### Bone marrow mononuclear cells

BMMNCs are isolated from bone marrow which was aspirated from the posterior iliac crest of patients under local anesthesia by density-gradient centrifugation. BMMNCs are cell populations composed of multiple stem/progenitor cells. The makers of BMMNCs consist of three types: endothelial lineage markers such as CD31 and vascular endothelial growth factor receptor (VEGFR), BMMSC surface markers, and HSC markers [51].

Bone marrow-derived CD11b^+^CD14^+^ monocytes improved liver fibrosis by reducing oxidative stress and inflammation, monocyte-treated mice had higher levels of glutathione (GSH) which represented antioxidant capacity. Moreover, the monocyte-treated group showed pro-inflammatory TNF-α and IL-6 downregulation and anti-inflammatory IL-10 upregulation [52]. Except for these, BMMNCs showed a significant decline in malondialdehyde and normalized total antioxidant capacity (TAC), glutathione peroxidase (GPx), catalase (CAT), and superoxide dismutase (SOD) [53].

### Endothelial progenitor cells

Endothelial progenitor cells (EPCs) are obtained from various tissue, most commonly referred to in clinical practice as BMMNCs. BMMNCs were induced under an endothelial complete medium consisting of cytokine vascular endothelial growth factor (VEGF), insulin-like factor-1 (IGF-1), basic fibroblast growth factor (bFGF), and epidermal growth factor (EGF). EPCs acquired in this way are positive for VEGFR-1, von Willebrand factor (vWF), and Ulex-binding [54].

EPCs transplantation activated HGF-mediated hepatocyte proliferation and increased sinusoidal blood vessel density by secreting higher MMP-2 and VEGF in carbon tetrachloride-induced liver fibrosis [55]. Unexpectedly, the results of one study indicated that the percentage of infused EPCs and collagen proportionate area was substantially increased in the fibrotic liver, and this may be attributed to higher levers of VEGF and TGF-β caused by EPCs activated hepatic stellate cells [56].

### Liver cells

The definition of liver stem cells remains controversial, as hepatocytes and bile duct epithelial cells can be regarded as their own respective specific stem cells in certain cases [57]. The liver cells currently used in clinical trials are adult or fetal hepatocytes. Hepatocytes are collected by the collagenase perfusion technique [58]. Fresh hepatocytes should be more than 60% viable and due to the specific characteristics of hepatocytes, isolated hepatocytes should be immediately transplanted or frozen for storage [16].

In liver cirrhosis or failure, hepatocyte transplantation aims to provide functional parenchymal support to the damaged mass, bridging the recovery of the liver [15]. In fact, hepatocyte transplantation not only is inefficient but also needs to suppress the patient's immune system. Transplantation of 3–5% liver mass only results in 0.5% engraftment of the host liver and grafted cell loss is due to patient’s CD8^+^ T-lymphocytes alloreactivity directed against a donor HLA antigen [59]. Currently, there is no standard immunosuppressive regimen for hepatocyte transplantation. Most centers adopt the same regimen as OLT, such as the combination of tacrolimus and monoclonal antibodies [16]. Strikingly, liver progenitor cells (LPCs) are a class of bipotent stem cells that can further differentiate into hepatocytes and bile duct epithelial cells; they are considered to play a crucial role in long-term liver injury [60]. LPCs are recognized as an essential chain of endogenous liver regeneration. Recent work shows that hepatocytes and bile duct epithelial cells can trans-differentiate between each other to promote liver repair without undergoing the state of hepatic progenitor cells during chronic liver injury [61, 62].

### Hepatocyte-like cells

Various stem cells have shown potential for hepatic differentiation in vivo, so hepatocyte-like cells (HLCs) have also been used clinically to treat liver fibrosis or cirrhotic disease. However, MSCs, mesoderm-derived cells, have been pending for their ability to differentiate into mature hepatocytes along the endoderm.

Safe and homogenous HLCs are mainly obtained by adding cytokines, which mimics the process of embryonic development of the mammalian liver by culturing cells in a medium supplemented with growth factors necessary for liver development [63]. For MSC, hepatic specification is performed by fibroblast growth factor 2 (FGF2), fibroblast growth factor 4 (FGF4), bone morphogenetic protein 4 (BMP4), and HGF. The mature stage often requires the application of oncostatin M (OSM), dexamethasone, and ITS premix [64]. Hepatic maturation can be monitored by the selective up-and down-regulation of a plethora of genes, early hepatic maturation markers such as α-fetoprotein (AFP), ALB, alpha-1-antitrypsin (A1AT), and so on, final hepatic maturation markers CytochromeP450 enzymes, hepatic transporters, and all the hepatic enzymes for urea cycle and more [65]. After hepatic differentiation, CD73 was down regulated, and MSC-derived HLCs have an upregulated ALB, A1AT, hepatocyte nuclear factor-4α (HNF4α), and a transient increase in AFP expression. In addition, it has also been reported that MSC-derived HLCs expressed CYP3A4, CYP1A2, MRP2, UTG1A6 [66], G-6-P [67], and other functional genes specific to hepatocytes. To our regret, many functions of MSC-derived HLCs are still only one percent or even one-thousandth of those of primary hepatocytes, depending on the mRNA expression level. The absence of HepPar1 and loss of hepatogenic phenotype suggested that MSCs had never differentiated into the mature hepatocytes [68].

The researchers compared the curative effect of the MSCs-treated group and the HLCs-treated group separately; liver function improved in both groups, but MSCs exhibited better results in some parameters [69, 70]. This may contribute to the fact that MSC-derived HLCs are often limited and differ functionally from primary hepatocytes [71]. In one study, differentiated induced pluripotent stem cells (iPSCs) were suggested to engraft more efficiently than undifferentiated iPSCs. Treatment with 4.0 × 10^7^ iPSCs-derived hepatocytes per kilogram of body weight significantly alleviated heavy liver failure in mice; yet, an equivalent dose of iPSCs had no significant therapeutic effect [72]. This suggested that embryonic stem cells (ESCs) or iPSCs need to be differentiated first to have a great therapeutic effect.

### Potential limitations and strengths of the various types of stem cells

In this section, we focused our attention and criticism on describing discrepancies and different potency in using stem cells from different sources. Table 2 describes different sources of stem cells along with their disadvantages and advantages in clinical application.

**Table 2:** Comparison of stem cell sources and their disadvantages and advantages

Source	Cell type	Disadvantages	Advantages
Bone marrow Blood	BMMSCs BMMNCs HSCs	Invasive acquisition Limited quantity Risk of infection Heterogeneity Delicate sorting Immunogenicity	Strong potential for differentiation Validated safety and efficacy in most clinical trials
Umbilical cord	UCMSCs	Cellular ageing after continuous passaging	No ethical concern Superior immunosuppression Low cost Highly proliferative
Adipose	ADMSCs	Moderated differentiation capacity	No ethical concern Autologous Available at large scale No age limit for the donor High isolation success and proliferation rate Resistant to oxidative stress-induced apoptosis and senescence
Menstrual blood	MenSCs	Inadequate clinical trials	Easy access Highly proliferative Genetic stability
Teeth	SHED	Absence of rigorous and systematic preparation protocol	Easy access Strong potential for differentiation
Liver	LDMSCs Liver cells	Invasive acquisition Difficult supply Hard to expand in vitro	Relative mature liver function Preferred differentiation into hepatocytes
In vitro	EPCs HLCs	Absence of a standard preparation protocol Variations between batches Limited quantity	No ethical concern Hepatocyte-like properties

Although UCMSCs for current clinical use are allogeneic, perinatal MSCs highly expressed HLA-G, which is essential in the adaptation of the maternal immune system during pregnancy as well as a suppressive effect on all immune cells, giving UCMSCs an innate immune privilege [73]. UCMSCs displayed the strongest proliferative capacity at P3, which then slowly diminished with increased passages in vitro [74]. UCMSCs characteristically expressed transcriptomes associated with neuronal development including NPY, this implies that UCMSCs are more preferentially differentiated into neural cells and have prospective applications in neurological disorders [75]. The low cost of preparation, the ease of large-scale expansion in vitro, and the absence of ethical concerns make UCMSCs the most promising source of cells for clinical use. Compared to UCMSCs, BMMSCs have a superior capacity for differentiation along the mesoderm including osteogenesis or chondrogenesis and inferior adipogenesis. A set of genes related to antimicrobial activity was more expressed in BMMSCs, while genes related to matrix remodeling were higher expressed in UCMSCs [76]. BMMSCs possess strong differentiation potential, but human BMMSCs are obtained by invasive means and quantities are limited, both of which hinder the further development of BMMSCs in clinical applications. Because ADMSCs are more resistant to hyperoxia-induced apoptosis, and oxidative stress-induced senescence, moreover, its superior proangiogenic capacity and high activity of telomerase, ADMSCs present a unique benefit over BMMSCs in terms of regenerative capacity [77]. There are no significant differences between ADMSCs and BMMSCs in their immunophenotype. Another most obvious merit of ADMSCs is their higher isolation success and proliferation rate [78]. ADMSCs are simple to harvest, widely available, and cell viability is not constrained by the age of the donor, so they have a wide scope in regenerative medicine. LDMSCs are more closely to the environment of the liver itself, especially as they express endoderm-related markers, but the normal fetal liver is of restricted sources. LDMSCs are not currently widespread in clinical trials. Compared with BMMSCs, MenSCs without induction stimuli are able to expand at 18 passages without chromosome abnormalities [37]. MenSCs have 2–4 fold higher proliferative capacity compared to BMMSCs, with 30–47 populations doubling before senescence [79]. The concern, however, is that there are few clinical trials using MenSCs for liver disease, standard isolation protocols, and culture conditions are crucial issues. Safety and efficacy have yet to be fully verified. SHED are more potent than BMMSCs in neurogenic [80]. SHED are accessible, easy isolation, and low invasive, but the absence of rigorous clarification on these signatures and efficacy will hinder their foreground.

HSC transplantation was first applied in the field of hemopathy. Maintaining the self-renewal and the undifferentiated state of HSCs for ex vivo expansion is currently a serious challenge considering the clinical application [81]. Shortage of HSC meeting clinical management criteria and limited source of low immunogenic HLA-matched HSCs [82]. Given the complexity and high cost of preparing autologous HSCs and the restricted quantity of cells, HSCs are more appropriate for patients with regenerative disabilities accompanied by hematological disabilities.

Because of the abundance of cell types contained in BMMNCs, BMMNCs have heterogeneity. BMMNCs are increasingly being used in clinical trials with no further subdivision. BMMSCs transplantation improved limb ischemia and increased blood flow, yet BMMNCs had no significant effect [83]. Nevertheless, due to the complex composition of BMMNCs, there is still ambiguity in their clinical application.

The key limitation of EPCs in clinical treatment is the low number and one possible solution is to use BMMNCs without pre-selection [84]. EPCs need to be artificially induced to grow in vitro, the heterogeneity of EPCs and the irregularity of their manufacture have hindered their clinical development.

Liver parenchymal cells are the most ideal source of cells for the treatment of liver disease, but PHHs-derived therapy has encountered many challenges in clinical application, such as the limited ability of PHHs to proliferate under traditional culture conditions, the loss of their original phenotype and function in vitro, and difficulty in the acquisition process [71]. These forced us to hunt for a cell source comparable to hepatocyte.

The induction of HLCs in vitro requires weeks-long cytokines irrigation. Moreover, the heterogeneity of the differentiated HLCs and the hepatic function of HLCs are closely dependent on the cell source, the induction protocol, and the batch of cytokines. Currently, a standardized manufacturing process and the number of cells available for the clinic are the disturbing issues we need to address.

## Mechanisms of stem cell therapy for liver fibrosis

**Fig. 3:**
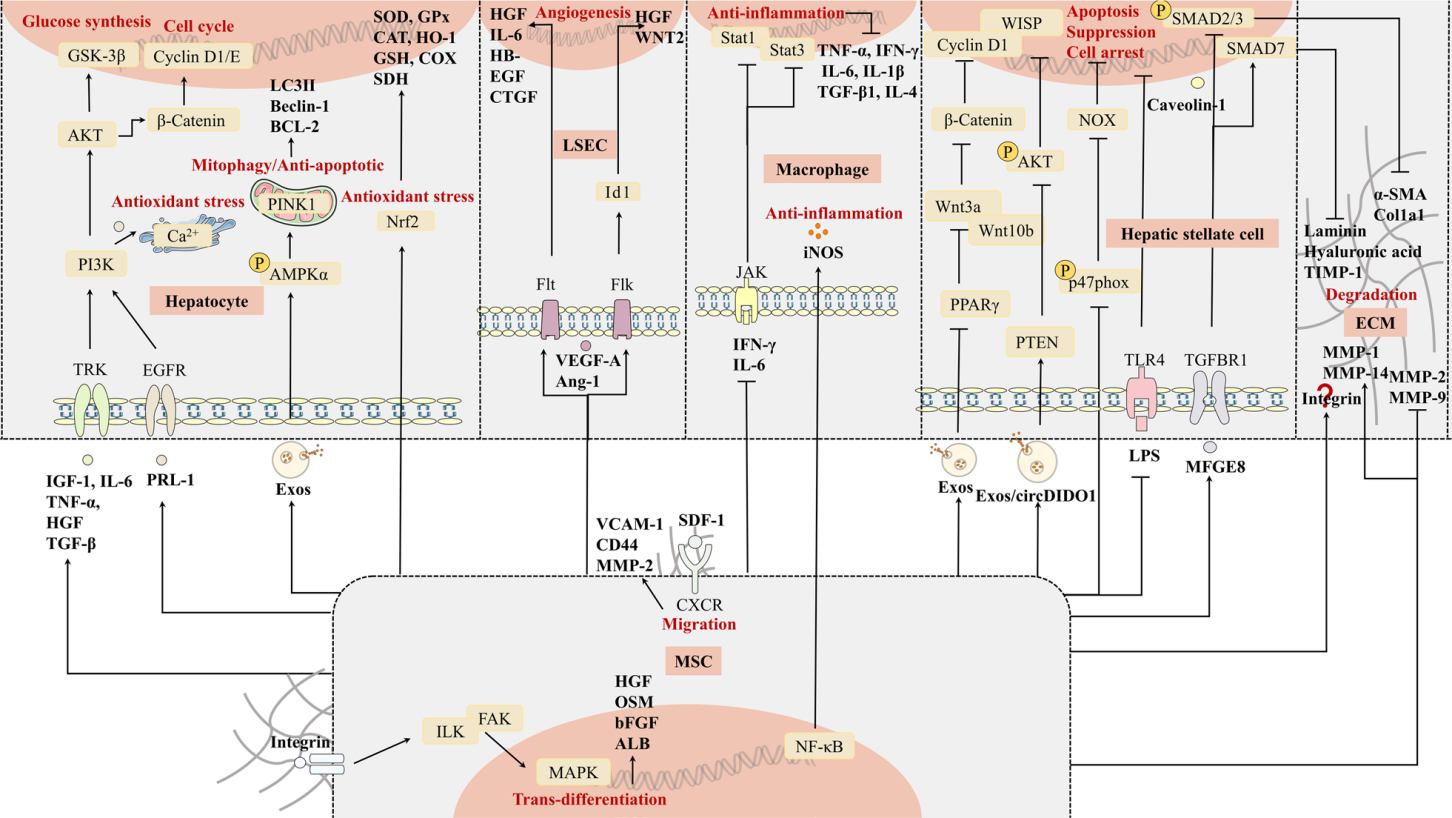
Mechanisms of stem cells for liver fibrosis. Stem cells, mainly MSCs, replace the hepatocyte function through migration and transdifferentiation, secrete a variety of cytokines to promote hepatocyte proliferation, enhance anti-apoptosis and anti-oxidation capacity of hepatocytes, promote LESC proliferation, inhibit the secretion of inflammatory factors in liver macrophages, repress the activation of hepatic stellate cells and promote the apoptosis of activated cells, crucially, degrade the ECM to delay the process of liver fibrosis. MSC, mesenchymal stem cell; SDF-1, stromal cell-derived factor-1; LSEC, liver sinusoidal endothelial cells; CXCR, chemokine receptor; VACM-1, vascular cell adhesion molecule-1; MMP-2, matrix metalloproteinases-2; HGF, hepatocyte growth factor; OSM, oncostatin M; bFGF, basic fibroblast growth factor; ALB, albumin; IGF-1, insulin-like factor-1; TRK, tyrosine kinase receptor; EGFR, epidermal growth factor receptor; IL-6, interleukin-6; TNF-α, tumor necrosis factor-α; TGF-β, transforming growth factor-β; PRL-1, phosphatase of regenerating liver-1; VEGF-A, vascular endothelial growth factor receptor-A; Ang-1, angiotensin-1; Flt1, Fms-like tyrosine kinase 1; Flk1, Fetal-like kinase 1; HB-EGF, heparin binding epidermal growth factor-like growth factor; CTGF, connective tissue growth factor; IFN-γ, interferon-γ; IL-1β, interleukin-1β; IL-4, interleukin-4; iNOS, nitric oxide synthase; GPx, glutathione peroxidase; CAT, catalase; SOD, superoxide dismutase; HO-1, hemeoxygenase-1; GSH, glutathione; COX, cytochrome-c oxidase; SDH, succinate dehydrogenase; Exos, exosomes; MFGE8, milk fat globule-EGF factor 8; TGFBR1, TGF-β type I receptor; LPS, lipopolysaccharides; JAK, Janus kinase; TLR4, toll-like receptor 4; α-SMA, alpha smooth muscle actin; Col1a1, Type I collage; TIMP-1, tissue inhibitor of metalloproteinase; MMP-1, matrix metalloproteinases-1; MMP-9, matrix metalloproteinases-9; MMP-14, matrix metalloproteinases-14; ECM, extracellular matrix

The development of liver fibrosis is a process of wound healing, critical events such as hepatocyte death, immune cell infiltration, liver injury, myofibroblast activation, and excessive deposition of the extracellular matrix are implicated. We will provide insight into these five aspects of how stem cells modulate (Fig. 3). Of interest is the fact that more than 30 years ago, mesenchymal stem cells referred to their multipotency and highly proliferative capacity [85]. But it is currently accepted that the importance to refine such multipotent cells as stromal medicinal products [86], whose paracrine action rather than differentiation capacity is leading to regenerative potential.

### Liver regeneration

The mechanisms by which stem cells promote liver regeneration can be subdivided into three aspects: migration of stem cells into the liver and transdifferentiation into hepatocytes, secretion of angiogenesis-related cytokines to promote neovascularization synergistically enhancing liver regeneration, and activation of proliferative signaling pathways in host endogenous hepatocytes, the most vital of these.

#### Migration and transdifferentiation

Chemokine ligand 12 (CXCL12), also known as stromal cell-derived factor-1 (SDF-1), is present in liver sinusoidal endothelial cells (LSECs), tumor cells, and hepatic stellate cells. When the liver is acutely or chronically damaged, SDF-1 is overexpressed in the liver [87]. It has been shown that CXCL12/chemokine receptor (CXCR) is a critical chemotactic signal that recruits MSC to the liver, and concentration gradients of SDF-1 significantly affect stem cell migration. SDF-1 can induce activation of α4β1 integrin in MSC, producing firm adhesion between MSC and endothelial cells [88]. SDF-1 binds to either CXCR4 or CXCR7 on the stem cells, with the CXCR7 pathway promoting liver regeneration but with the CXCR4 pathway promoting fibrosis [89]. In recent years, it has also been found that CXCR7, although expressed at low levels on the cell surface, has a ten-fold greater binding capacity to CXCL12 than CXCR4. Under hypoxic conditions and during hepatic differentiation, CXCR7 overexpression significantly increases the migratory capacity of MSC by upregulating the levels of vascular cell adhesion molecule-1 (VCAM-1), CD44, and MMP-2, which promote the adhesion of MSC by their interaction with the ECM [90].

After MSC homed the damaged liver, liver-specific ECM switched hepatogenesis of MSC via integrin pathway and downstream focal adhesion kinase (FAK) and integrin-linked kinase (ILK) which upregulated the expression of MAPK but not affected the expression of AKT, resulting in a significant increase of hepatogenic molecules such as HGF, OSM and bFGF in MSC. Then, there was a significant increase in the number of ALB-positive MSC in the presence of liver-specific ECM [30].

#### Hepatocyte proliferation

However, there is the controversy that the therapeutic effect of MSC is due to the cytokines they secrete to activate the facultative pathway of liver cells rather than their ability to transform into hepatocytes to replace damaged liver parenchymal cells [91–93]. Intravenously injected MSC increased serum IGF-1 level, IGF-1 acts on insulin-like factor 1 receptor (IGF1R) and upregulates downstream PI3K/AKT pathway [94]. MSC-conditioned medium (CM) can significantly induce gene expression of IL-6, TNF-α, HGF, and TGF-β, which are closely related to hepatocyte proliferation [95]. These cytokines interact with tyrosine kinase receptors to activate the PI3K/AKT signaling pathway [96]. AKT plays an important role in cell growth, proliferation, and glucose metabolism. MSC transplantation resulted in improved glucose synthesis through AKT/GSK-3β pathway. Meanwhile, MSC promotes hepatocyte proliferation mainly via the AKT/β-Catenin pathway. Cyclin D1 and Cyclin E, downstream proteins of β-Catenin, accelerated the G1/S phase in the cell cycle after MSC transplantation [97, 98].

#### Angiogenesis

The angiogenesis process is also involved in promoting liver regeneration. Condition medium derived from MSC treatment caused upregulation of the proangiogenic factors VEGF-A, angiotensin-1 (Ang-1), and receptors VEGF-R1 (Fms-like tyrosine kinase 1; Flt1) and VEGF-R2 (Fetal-like kinase 1; Flk1) [99]. Meanwhile, the number of regenerated microvessels was enhanced by administrating with MSC-derived exosomes [100]. Activation of VEGF-A signaling promoted liver cell proliferation through an increased blood supply and the release of paracrine factors. VEGF-A/Flt1 signaling promoted the proliferation of hepatic cells through LSEC-derived secreted factors, such as HGF, IL-6, and heparin binding epidermal growth factor-like growth factor (HB-EGF), connective tissue growth factor (CTGF). MSC treatment resulted in hepatocyte proliferation and neovascularization through hepatic growth mediators WNT2 and HGF secreted by LSEC in the VEGF-A/Flk1/Id1 signaling pathway [99, 101].

### Immunoregulation

**Fig. 4:**
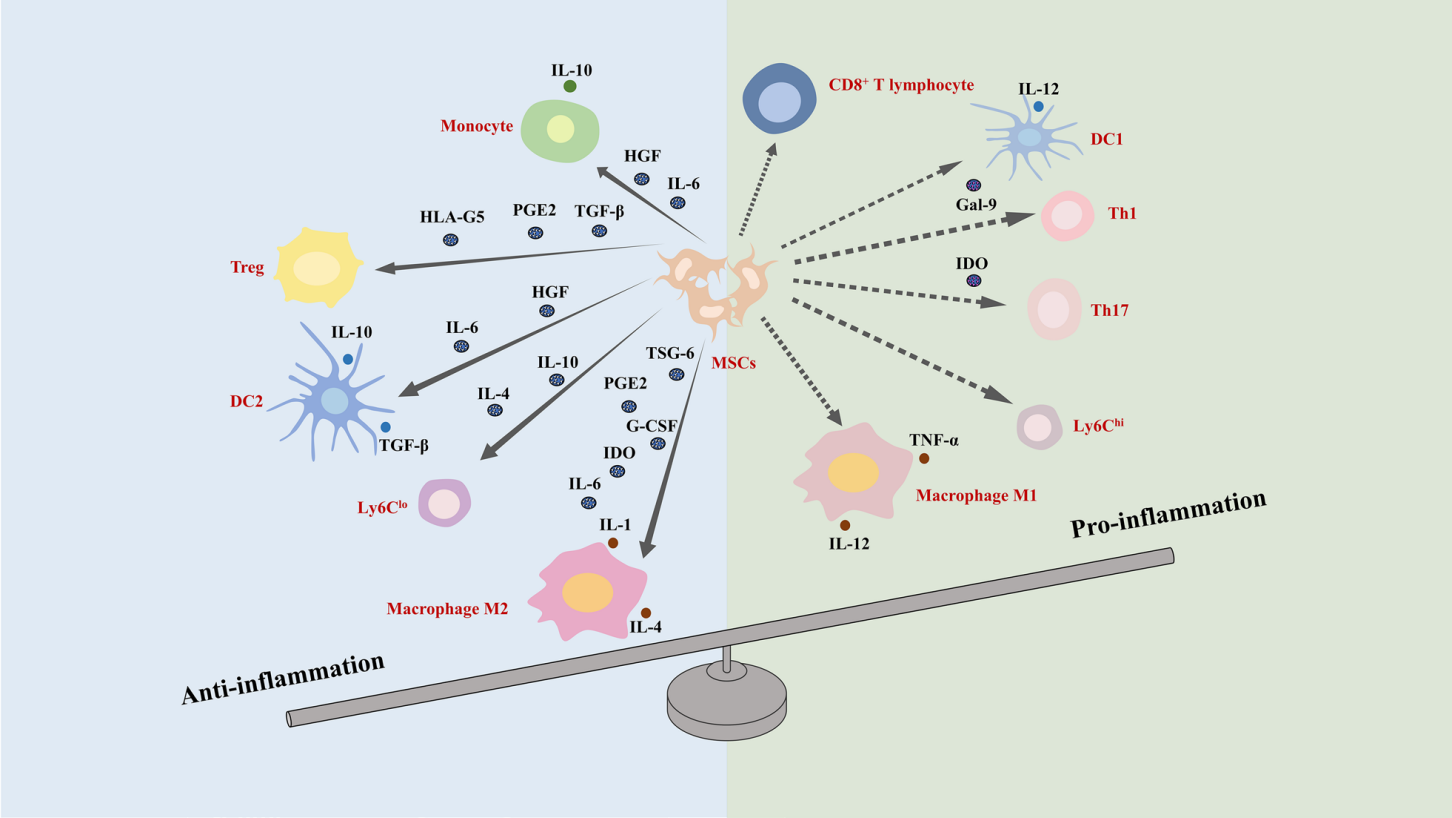
MSC-mediated immunoregulation in liver fibrosis. MSCs secrete different cytokines that regulate the transition of immune cells in the liver from a pro-inflammatory phenotype to an anti-inflammatory phenotype. The solid line represents the promotion, and the dashed line represents the inhibition. MSC, mesenchymal stem cell; IL-6, interleukin-6, HGF, hepatocyte growth factor; IL-10, interleukin-10, IL-4, interleukin-4; PGE2, prostaglandin E2, TSG-6, TNFα simulated gene/protein 6; G-CSF, granulocyte-colony stimulating factor; IDO, indolamine 2,3-dioxygenase; DC1, type 1 dendritic cell; DC2, type 2 dendritic cell; Th1, T-helper 1; Th17, T-helper 17; Treg, regulatory T cell; Gal-9, galectin-9; HLA-G5, human leukocyte antigen-5; TGF-β, transforming growth factor-β

MSC can initiate immunosuppression through both intercellular contacts and secreted signaling molecules, and the immunosuppressive microenvironment is formed mainly by MSC transforming various immune cells to maintain a tolerant state (Fig. 4).

In the presence of IL-6 and HGF secreted by UCMSC, monocytes are suppressed differentiation toward other immune cell types and it is more inclined to develop a protective IL-10-producing phenotype [102]. Macrophage is known to be an important target of MSC in the regulation of intrinsic immunity. MSC skew the polarization of macrophages M2 expressing the anti-inflammatory mediators IL-1 and IL-4 and reduces the production of pro-inflammatory macrophages M1 expressing the inflammatory signals IL-12 and TNF-α by secreting soluble molecules such as prostaglandin E2 (PGE2), TNF-α stimulated gene/protein 6 (TSG-6), IL-6, G-CSF [103], and indolamine 2,3-dioxygenase (IDO) [104]. The increase in the ratio of M2 polarization to M1 polarization is mediated by PTEN/AKT signaling pathway activated by MSC-derived exosomes [105]. Besides, BMMSCs converted phenotypic switch from pro-fibrotic Ly6C^hi^ subpopulation to protective Ly6C^lo^ subset in the presence of IL-4 and IL-10 [106]. Furthermore, under the effects of IL-6 and HGF secreted from MSC, mature type 1 dendritic cells (DC1s) are prone to differentiation toward tolerogenic type 2 dendritic cells (DC2s) via the phosphorylation of AKT which increases the expression level of IL-10 and TGF-β while decrease secretion of IL-12 [107]. Interestingly, hepatic stellate cells are antigen-presenting cells, and inhibition of hepatic stellate cells activation can reduce the adaptive immune response they trigger.

In the adaptive immune response, MSC abrogates the activation and proliferation of both CD4^+^ T-helper (Th) and CD8^+^ cytotoxic T-lymphocytes [108]. Additionally, MSC downregulated T-helper 1 (Th1) through upregulation of galectin-9 (Gal-9) [109], suppressed T-helper 17 (Th17) cells in IDO-dependent manner [110], and induced regulatory T cells (Tregs) through activation of Notch1 pathway or secretion of human leukocyte antigen-5 (HLA-G5), PGE2 and TGF-β [104].

Recently, MSC secreted IL-2 receptor (IL-2R) may act as a decoy receptor to inhibit IL-2 activity, IL-2R has attracted much attention as a new immune mediator in the regulation of MSC involvement [103].

### Resistance to liver injury

Hepatic fibrosis is exposed to chronic damage over a long period, a multitude of molecules such as pro-inflammatory factors, oxidative stress products, apoptotic mediators, and other cytokines can affect its progression. MSC transplantation offers anti-inflammatory, anti-oxidant, and anti-apoptotic benefits.

#### Anti-inflammatory

MSC transplantation significantly reduced the level of pro-inflammatory factors TNF-α, interferon-γ (IFN-γ), IL-6, IL-1β, TGF-β1, and IL-4 in the liver by downregulating the excessive activated IFN-γ/Stat1 and IL-6/Stat3 signaling pathways. TGF-β1, secreted by liver macrophages, is the main molecule for hepatic stellate cells activation [111]. HNF-4α overexpressing MSC exerted anti-inflammatory effects by enhancing the expression of inducible nitric oxide synthase (iNOS) in MSC via activation of the NF-κB pathway [112].

#### Anti-oxidant

Oxidative phosphorylation and liposome peroxidation in hepatic stellate cells and macrophages produce large amounts of NADPH oxidases (NOXs), which are one of the components mediating reactive oxygen species (ROS), reactive nitrogen species (RNS) or ROS regulate the proliferation and pro-inflammatory phenotype of hepatic stellate cells mainly through the NF-κB pathway [113]. UCMSC transplants reduce malondialdehyde (MDA), the major product of lipid peroxidation in mitochondria, by secreting antioxidant enzymes such as SOD, GPx, CAT, hemeoxygenase-1 (HO-1), and GSH [114]. It also restores normal mitochondrial function by increasing the levels of cytochrome-c oxidase (COX) and succinate dehydrogenase (SDH) in the mitochondrial ATPase and respiratory chain. UCMSC reduces the release of oxidative stress mediators by inducing antioxidant responses through upregulation of the Nrf2/HO-1 pathway. Nrf2 plays an important role in maintaining the structural integrity and functional stability of mitochondria [115]. Phosphatase of regenerating liver-1 (PRL-1)-overexpressing MSC decreased endoplasmic reticulum (ER) stress via PRL-1 binding to epidermal growth factor receptor (EGFR), thereby activating PI3K expression finally regulating the release of Ca^2+^ in hepatic cells [116].

#### Anti-apoptotic

Engrafted BMMSC underwent apoptosis in fibrotic tissue. Hepatic stellate cells are prime candidates for responding to apoptotic cells in the liver, phagocytosis of apoptotic bodies leads to profibrogenic phenotype in hepatic stellate cells [117]. Interestingly, with the employment of the PtdSer/MerTK/ERK axis, Ly6C^lo^ macrophages engulfed apoptotic bodies by secreting MMP-12 [106]. BMMSC-derived exosomes (BMMSC-Exos) can be taken up by hepatocytes and entered the cytoplasm. Treatment with BMMSC-Exos or hUCMSCs reduced hepatocyte apoptosis by producing protective autophagosomes in liver cells. Treatment with hUCMSC increased PINK1-dependent mitophagy in hepatocytes through AMPKα phosphorylation [118]. After the application of BMSC-Exos, the expression levels of autophagy marker proteins LC3II, Beclin-1, and anti-apoptotic protein BCL-2 were significantly upregulated, while the expression levels of proapoptotic protein caspase-3 and Bax were downregulated [119].

### Suppression of myofibroblast activity

The appearance of myofibroblasts in the liver is closely related to fibrosis. Myofibroblasts have a pro-fibrogenic and pro-inflammatory phenotype and are highly expressed α-SMA and TIMP-1 [113]. Bone marrow, fibroblasts in circulation, hepatocytes, and bile duct cells can be transformed into myofibroblasts through epithelial-mesenchymal transition (EMT); however, activated hepatic stellate cells are most common [120]. Hepatic stellate cells are specific cells that are normally quiescent, and they are activated when the liver is damaged, transforming into myofibroblasts. Activation of hepatic stellate cells is initiated by various mediators such as the above pro-inflammatory factors, oxidative stress mediators, inflammatory stimuli generated by apoptosis or necrosis of liver parenchymal cells, then the activated hepatic stellate cells secrete various pro-inflammatory factors that exacerbate the inflammatory response in the liver [113, 121]. Blocking myofibroblasts is a key target for the treatment of fibrosis.

TGF-β is considered to be one of the most vital signals involved in stellate cell activation. Some studies have shown that milk fat globule-EGF factor 8 (MFGE8), one of the secretomes from MSC, is an anti-fibrotic protein that inhibited hepatic stellate cells activation by downregulating TGF-β type I receptor (TGFBR1) [122]. Caveolin-1, which is a potential target for MSC therapy, showed a significant suppression effect on hepatic stellate cells. On the one hand, the phosphorylation of SMAD2, a key link in the TGF-β/SMAD axis, was reduced by upregulated Caveolin-1. On the other hand, Caveolin-1 in activated hepatic stellate cells was restored [123, 124]. BMMSC-Exos declined the expression of Wnt pathway-associated proteins such as peroxisome proliferator-activated receptor γ (PPARγ), Wnt3a, Wnt10b, β-catenin, and most critical downstream WISP1 and Cyclin D1 in hepatic stellate cells, leading to inhibit hepatic stellate cells activation [125]. Amnion-derived MSC (AMSC) was displayed to repress hepatic stellate cells activation by inhibiting earlier steps of the LPS/TLR4 pathways but not downstream NF-κB transcriptional activity [126]. MSC-originated exosomal circDIDO1 sponged miR-143-3p in hepatic stellate cells, causing cell cycle arrest, suppression, and apoptosis through promoting PTEN and repressing the ratio of p-AKT/AKT [127]. More, BMMSC transplantation significantly reduced the NOXs which are vital intermediates in the motivation of hepatic stellate cells through decreasing phosphorylation of p47phox, a ligand of NOX [128]. Besides, UCMSC-Exos increased the expression of the epithelium-associated marker E-cadherin while decreasing N-cadherin- and vimentin-positive cells, thereby inhibiting EMT [129].

### Extracellular matrix degradation and remodeling

Liver fibrosis is associated with both excessive deposition of ECM and reduced activities of the ECM lysis. ECM degradation and remodeling have been considered a key step in reversing liver fibrosis and limiting the advancement of liver fibrosis to cirrhosis.

TGF-β remains a classic cytokine that participates in the regulation of ECM, BMSC transplantation significantly downregulated the mRNA expression of SMAD3 downstream of TGF-β1 and TGFBR1 in myofibroblasts and increased the mRNA levels of SMAD7. Likewise, hUCMSC treatment significantly suppressed the expression of SAMD2 protein [25]. SMAD2 and SMAD3 mediate the transcription of pro-fibrotic factor α-SMA or Col1a1, whereas SMAD7 has a negative anti-fibrotic regulatory effect [130]. BMSC overexpressing Smad7 significantly reduced plasma levels of laminin and hyaluronic acid, components of the ECM. Furthermore, Smad7-MSC mediated an increase in serum expression of MMP-1 and a decrease in TIMP-1 [131]. MMP is a class of matrix-degrading proteases that mainly degrade scarring in the subendothelial space. MMP-1 is a member of the MMP family and is responsible for the degradation of matrix type I collagen (Col1a1) and type III collagen (Col3a1). In contrast, TIMP inhibits MMP activity by forming a reversible covalent complex with the corresponding MMP. IC-2-engineered MSC sheets reverse established liver fibrosis by augmenting the secretion of activated MMP-1 and MMP-14, particularly MMP-14, although generally considered to be a membrane-bound protein but has been proven to act in a soluble form as well. MMP-14 can activate MMP-2 and MMP-13, which are mainly involved in the dissolution of Col1a1 [132]. Exogenously administered MSC protects against fibrosis by decreasing p-STAT3 levels and thereby reducing STAT3-dependent MMP-9 production [133]. It is noteworthy that MMP-9, unlike traditional MMPs, can stimulate the EMT process and thus exacerbate fibrosis. In the liver fibrosis model, the expression of MMP-9 was higher than in the normal group, probably due to the synergy between MMP-9 and MMP-2 in restoring the structural integrity of liver cells and maintaining the normal morphology of the basement membrane of the liver sinusoids. MMPs cleave matricellular proteins at specific sites in the ECM to produce new active products and regulate tissue matrix attachment is a current hot topic of interest.

In parallel to reducing deposited ECM, MSC can also mediate the rearrangement of ECM to improve fibrosis. MSC rescue established fibrosis by reducing mRNA for integrin, which acts as a bridge between various extracellular components such as fibronectin, fibrinogen, laminin, and intracellular actin, mediating the architecture and adhesion of the ECM [134]. ILK-modified MSC conditioned medium inhibits fibrosis by reducing Col1a1 and Col3a1, TIMP, and CTGF. ILK interacts with the structural domains of intracytoplasmic integrins to regulate integrin-related functions, however, the exact mechanism by which ILK-MSC transplantation acts on integrins to cause matrix rearrangement remains unexplained [135].

MSC could improve fibrosis by downregulating or blocking the expression of secreted Frizzled-related protein 2 (sFRP2) and its downstream target Axin2, as well as increasing angiogenesis, suggesting that sFRP2 may be a potential target of action in MSC for the treatment of fibrotic disease models [136].

### EVs-driven mechanisms of action

Extracellular vesicles (EVs) are vesicles that form after the cell cargoes have sprouted. EVs have similar trophic and modulatory paracrine effects to stem cell-based therapy, but different mechanism of action and half-life.

The mechanism of action of EVs is currently based on two cargoes: RNA (especially miRNA) and protein. Toh et al. [137] suggested that due to the inadequate quantity of pre-miRNA in EVs, EVs are more likely to function through a protein-based network. A qualification is that the biological effect is largely based on the catalytic activity of enzymes, whereas some structural proteins in EVs are not able to induce a similar response. The uptake of EVs into recipient cells is via the binding of their lipids, carbohydrates, and some specific peptides to cell surface receptors. Uptake is only the first step; the details of how the contents of EVs are released into the cytoplasm of the recipient's cells are still unclear. Possible explanations are membrane fusion and release of cargo. Variant surface glycoprotein-G (VSG-G) present on the surface of EVs can mediate membrane fusion [138].

The fate of EVs in vivo can be divided into two phases, the first is rapid redistribution of EVs in various organs after systemic administration, with high doses of EVs first distributed in the spleen and liver, organs rich in vascularity. And the second phase is the final clearance of EVs from the body, the decay of the signal in blood followed a two-phase exponential decline, with a short half-life of 20 min and a longer half-life of more than 3 h [139]. With this in mind, locally administrated EVs could achieve higher concentrations in a target cell.

## Conclusions and future prospects

We summarize the current sources of stem cells available for clinical trials in cirrhosis and liver fibrosis from the perspective of clinical progress, the clinical phases, and the administration of stem cells. As a matter of course, the limitations and prospects of the various types of stem cells for clinical application are discussed. To further elucidate how stem cells perform in vivo, we present an in-depth exploration of stem cells improve liver fibrosis from liver regeneration, immunoregulation, resistance to liver injury, inhibition of myofibroblast activity, and extracellular matrix degradation and remodeling five aspects, demonstrating the therapeutic potential of stem cells.

In the mechanism of stem cell therapy for liver fibrosis, we note that the stem cells themselves may not serve a major function, conversely, paracrine mediators are thought to be pivotal to the action of stem cells. This gave birth to the conception of cell-free therapy. Cell-free strategies have been proposed without the potential for tumorigenicity, embolism, high cost of cell preservation, or possible exogenic infections. Stem cells secrete many soluble molecules such as nucleic acids, proteins, and lipids into the CM, they are encapsulated in EVs [140]. Hirata et al. [141] injected SHEDs and CM obtained from SHEDs into mice with liver fibrosis, and the results showed that both can improve fibrosis. CM targets multiple points of fibrosis development to achieve better outcomes. ADSCs were engineered to overexpress miRNA-181-5p to selectively transfer exosomes to damaged liver fibrosis, this suggests that EVs can act as a vehicle for the transport of MSC-secreted effective molecules and that genetically engineered EVs can achieve targeted delivery and amplified performance [142]. Regrettably, however, none of the clinical trials involved the use of stem cell-derived EVs for the treatment of liver fibrosis and cirrhosis. This is largely attributable to the standardized method for the extraction of large quantities of EVs or exosomes that has not been established and the dose and half-life of EVs remain unclear.

In contrast to cell-free therapies, cell-based pre-treatment is a hot topic for future research. Primary stem cells or unpretreated cells often fail to achieve the desired therapeutic effect due to poor homing ability, the very low survival rate after transplantation, and cellular senescence or decline in viability during in vitro culture. Enhancement strategies, including physiological microenvironment and pathological microenvironment to stimulate stem cell activation, have caused widespread concern [143]. A shift in the culture model is a common physiological way to enhance MSC. Compared with 2D cultured MSCs, 3D spheroids displayed a better capacity for antifibrosis both in vitro and in vivo [144]. Pathological pre-treatment includes hypoxia, inflammatory factors, bioactive compounds, and disease-associated cells or patient serum. IFN-γ pre-treatment and 3D culture of MSC showed increased production of both growth and immunoregulatory factors. Furthermore, these modified MSCs strongly reduced M1-induced inflammation [145]. Pre-activation of in vitro cultured MSCs with patient serum allows MSCs to have specific host-related functions and properties, thus allowing for individualized management strategies. The significant decrease in the infarct area, brain lesion, and apoptotic cells but the increase in neurogenesis positive cells number and trophic factors appeared in the stroke serum pretreated MSCs compared to the normal serum pretreated [146].

Integrated cell activation and cell-free therapy, Takeuchi et al. [147] found that EVs derived from IFN-γ pre-conditioned cells were more effective in relieving inflammation and fibrosis than the EVs group, signifying that IFN-γ altered the contents of EVs resulting in enhanced repair.

## Data Availability

All the data are included in the article.

## Abbreviations

ESLD: End-stage liver disease
OLT: Orthotropic liver transplantation
PHHs: Primary human hepatocytes
ECM: Extracellular matrix
MMP: Matrix metalloproteinase
TIMP: Tissue inhibitor of metalloproteinase
MSC: Mesenchymal stem cell
MELD: Model for end-stage liver disease
ALB: Albumin
ALT: Alanine aminotransferase
TBil: Total bilirubin
UCMSC: Umbilical cord mesenchymal stem cell
BMMSC: Bone marrow mesenchymal stem cell
HSC: Hematopoietic stem cell
GMP: Good manufacturing practice
HGF: Hepatocyte growth factor
PCNA: Proliferating cell nuclear antigen
PAK2: P21-activated kinase-2
BMMNC: Bone marrow mononuclear cell
IL: Interleukin
IGFBP-2: Insulin-like growth factor binding protein-2
IL-1Ra: IL-1 receptor antagonist
ADMSC: Adipose-derived mesenchymal stem cell
LDMSC: Liver-derived mesenchymal stem cell
MenSC: Menstrual blood-derived mesenchymal stem cell
SSEA4: Stage-specific embryonic antigen 4
α-SMA: Alpha smooth muscle actin
TGF-β1: Transforming growth factor-β1
MCP-1: Monocyte chemoattractant protein-1
SHED: Stem cells from human exfoliated deciduous teeth
TNF-α: Tumor necrosis factor-α
G-CSF: Granulocyte-colony stimulating factor
BMSC: Bone marrow stem cell
VEGFR: Vascular endothelial growth factor receptor
GSH: Glutathione
TAC: Total antioxidant capacity
GPx: Glutathione peroxidase
CAT: Catalase
SOD: Superoxide dismutase
EPC: Endothelial progenitor cell
vWF: Von Willebrand factor
VEGF: Vascular endothelial growth factor
IGF-1: Insulin-like factor-1
bFGF: Basic fibroblast growth factor
EGF: Epidermal growth factor
LPC: Liver progenitor cell
HLC: Hepatocyte-like cell
FGF2: Fibroblast growth factor 2
FGF4: Fibroblast growth factor 4
BMP4: Bone morphogenetic protein 4
OSM: Oncostatin M
AFP: α-Fetoprotein
A1AT: Alpha-1-antitrypsin
HNF4α: Hepatocyte nuclear factor 4α
ESC: Embryonic stem cell
iPSC: Induced pluripotent stem cell
CXCL12: Chemokine ligand 12
SDF-1: Stromal cell-derived factor-1
LSEC: Liver sinusoidal endothelial cell
CXCR: Chemokine receptor
VACM-1: Vascular cell adhesion molecule-1
FAK: Focal adhesion kinase
ILK: Integrin-linked kinase
IGF1R: Insulin-like factor 1 receptor
CM: Conditioned medium
Ang-1: Angiotensin-1
Flt1: Fms-like tyrosine kinase 1
Flk1: Fetal-like kinase 1
HB-EGF: Heparin binding epidermal growth factor-like growth factor
CTGF: Connective tissue growth factor
PGE2: Prostaglandin E2
TSG-6: TNFα simulated gene/protein 6
IDO: Indolamine 2,3-dioxygenase
DC1: Type 1 dendritic cell
DC2: Type 2 dendritic cell
Th: T-helper
Th1: T-helper 1
Th17: T-helper 17
Gal-9: Galectin-9
Treg: Regulatory T cell
HLA-G5: Human leukocyte antigen-5
IL-2R: IL-2 receptor
IFN-γ: Interferon-γ
iNOS: Inducible nitric oxide synthase
NOXs: NADPH oxidases
ROS: Reactive oxygen species
RNS: Reactive nitrogen species
MDA: Malondialdehyde
HO-1: Hemeoxygenase-1
COX: Cytochrome-c oxidase
SDH: Succinate dehydrogenase
PRL-1: Phosphatase of regenerating liver-1
ER: Endoplasmic reticulum
EGFR: Epidermal growth factor receptor
BMMSC-Exos: BMMSC-derived exosomes
EMT: Epithelial-mesenchymal transition
MFGE8: Milk fat globule-EGF factor 8
TGFBR1: TGF-β type I receptor
PPARγ: Peroxisome proliferator-activated receptor γ
AMSC: Amnion-derived mesenchymal stem cell
Col1a1: Type I collagen
Col3a1: Type III collagen
sFRP2: Secreted Frizzled-related protein 2
EVs: Extracellular vesicles
VSG-G: Variant surface glycoprotein-G
